# Facile synthesis of TiO_2_/ZrO_2_ nanofibers/nitrogen co-doped activated carbon to enhance the desalination and bacterial inactivation *via* capacitive deionization

**DOI:** 10.1038/s41598-017-19027-w

**Published:** 2018-01-11

**Authors:** Ahmed S. Yasin, Ibrahim M. A. Mohamed, Hamouda M. Mousa, Chan Hee Park, Cheol Sang Kim

**Affiliations:** 1 Department of Bionanosystem Engineering, Chonbuk National University, Jeonju, Jeonbuk 561-756 Republic of Korea; 2 Division of Mechanical Design Engineering, Chonbuk National University, Jeonju, Jeonbuk 561-756 Republic of Korea; 3 Chemistry Department, Faculty of Science, Sohag University, Sohag, 82524 Egypt; 4 Department of Engineering Materials and Mechanical Design, Faculty of Engineering, South Valley University, Qena, 83523 Egypt

## Abstract

Capacitive deionization, as a second generation electrosorption technique to obtain water, is one of the most promising water desalination technologies. Yet; in order to achieve high CDI performance, a well-designed structure of the electrode materials is needed, and is in high demand. Here, a novel composite nitrogen-TiO_2_/ZrO_2_ nanofibers incorporated activated carbon (NACTZ) is synthesized for the first time with enhanced desalination efficiency as well as disinfection performance towards brackish water. Nitrogen and TiO_2_/ZrO_2_ nanofibers are used as the support of activated carbon to improve its low capacitance and hydrophobicity, which had dramatically limited its adequacy during the CDI process. Importantly, the as-fabricated NACTZ nanocomposite demonstrates enhanced electrochemical performance with significant specific capacitance of 691.78 F g^−1^, low internal resistance and good cycling stability. In addition, it offers a high capacitive deionization performance of NACTZ yield with electrosorptive capacity of 3.98 mg g^−1^, and, good antibacterial effects as well. This work will provide an effective solution for developing highly performance and low-cost design for CDI electrode materials.

## Introduction

The limited availability of fresh water is one of the prime challenges in the current era, although water is the greatest widespread substance could be found in the environment^1,2^. Recently, water has presented a major dilemma for the sustainable development of many regions and work industries since increasing socio-economic development has brought ever more stringent conflicts between the growing demand for fresh water and reduction in supply, in addition to the high proportions of biocontamination. The most straightforward solution to improve the quantity, as well as the quality of water, is desalination of salty water source like sea or brackish water, based on the generality of Earth’s water being saline^3^. Several conventional desalination methodologies have been carried out to improve the supply of fresh water, the most common of which are reverse osmosis (RO), electrodialysis and thermal distillation^4,5^. Unfortunately, each of these current methods has experienced problems such as excessive operational cost, high energy consumption, and secondary pollution^6,7^. Investigations into other desalination techniques are becoming urgent in order to build economical and sustainable water systems^8^. Capacitive deionization (CDI) has emerged as an electrosorption desalination technique because of its competence in meeting several issues such as low power requirement, non-secondary pollution, inexpensive maintenance and operation, eco-friendliness, absence of membrane, and appropriateness for small-scale portable operation^9–11^. It is worth mentioning that the concept of CDI is similar to that of energy storage in supercapacitors^12^. In CDI, a pair of electrodes is controlled by a cell voltage (0.8–1.2 V), and meanwhile the potential is applied so that the salt ions can be trapped and stored in electric double-layer capacitors (EDLC) at porous carbon electrodes, which is the charging process. After some time, the applied voltage is set to zero or reversed, and ions are flushed out from the electrodes to the bulk solution^8,13–15^.

Hence, on the basis of the above mechanism the desired efficiency of a capacitive deionization depends mainly on the electrode performance, so intense research efforts have been funded to develop suitable electrode materials. The key characteristics that are considered as crucial in the electrode material for the production of practical CDI should involve high surface area, high conductivity, robust chemical inertia, high capacitance and good mechanical strength. Carbonaceous materials with high specific surface area such as graphene^16,17^, carbon nanofibers^18–20^, carbon aerogels^21^, carbon nanotubes^22^, ordered mesoporous carbon (OMC)^12,23,24^, and activated carbon (AC)^25,26^ have been utilized to improve the desalination efficiency, as well as modify the operational conditions. Recently, numerous novel electrode materials have been rationally fabricated and employed with several distinct features for significantly efficient capacitive deionization, such as nitrogen-doped porous carbon^27^ and 3D hierarchical carbon^28^. However, among these various carbon materials utilized, activated carbon stands out as one of the best candidates for electrode material in the CDI process, because of its markedly high surface area, superior electrical conductivity and cost-efficiency for scaling-up application^29^. In spite of the many significant advantages of AC, some shortcomings such as limited surface wettability and low specific capacitance additionally constrain AC by its limited rate of capability, due to the difficulty in their ion paths resulting in tardy transportation of the electrolyte ions during the charge-discharge process^30^. Consequently, numerous research efforts have focused on overcoming the above-mentioned shortcomings by hybridizing the EDLC of carbonaceous materials with the anchoring of inorganic materials (such as ZnO and SnO_2_) nanoparticles to elevate the specific capacitance and achieve high operating potential^31^. Specifically, TiO_2_ and ZrO_2_ have revealed promise as high efficient electrodes for CDI, due to their large electrical conductivity, superior hydrophilic properties, effectiveness in killing some microorganisms, and cheapness^32,33^. In this regard, the combination of TiO_2_ and ZrO_2_ is expected to be an efficient electrode of CDI^34^.

In recent years, nitrogen-doping has been considered one of the most favorable strategies to improve the desalination capacity of carbon material^35^. Compared to carbon, nitrogen-doped carbon has exhibited a significant effect with metal oxide because of its extraordinary charge polarization, which results from the different electronegativity between nitrogen and carbon, and which leads to superior electron transport properties. Additionally, nitrogen-doping could improve the conductivity and electrochemical performance as well as the wettability^36^.

**Figure 1:**
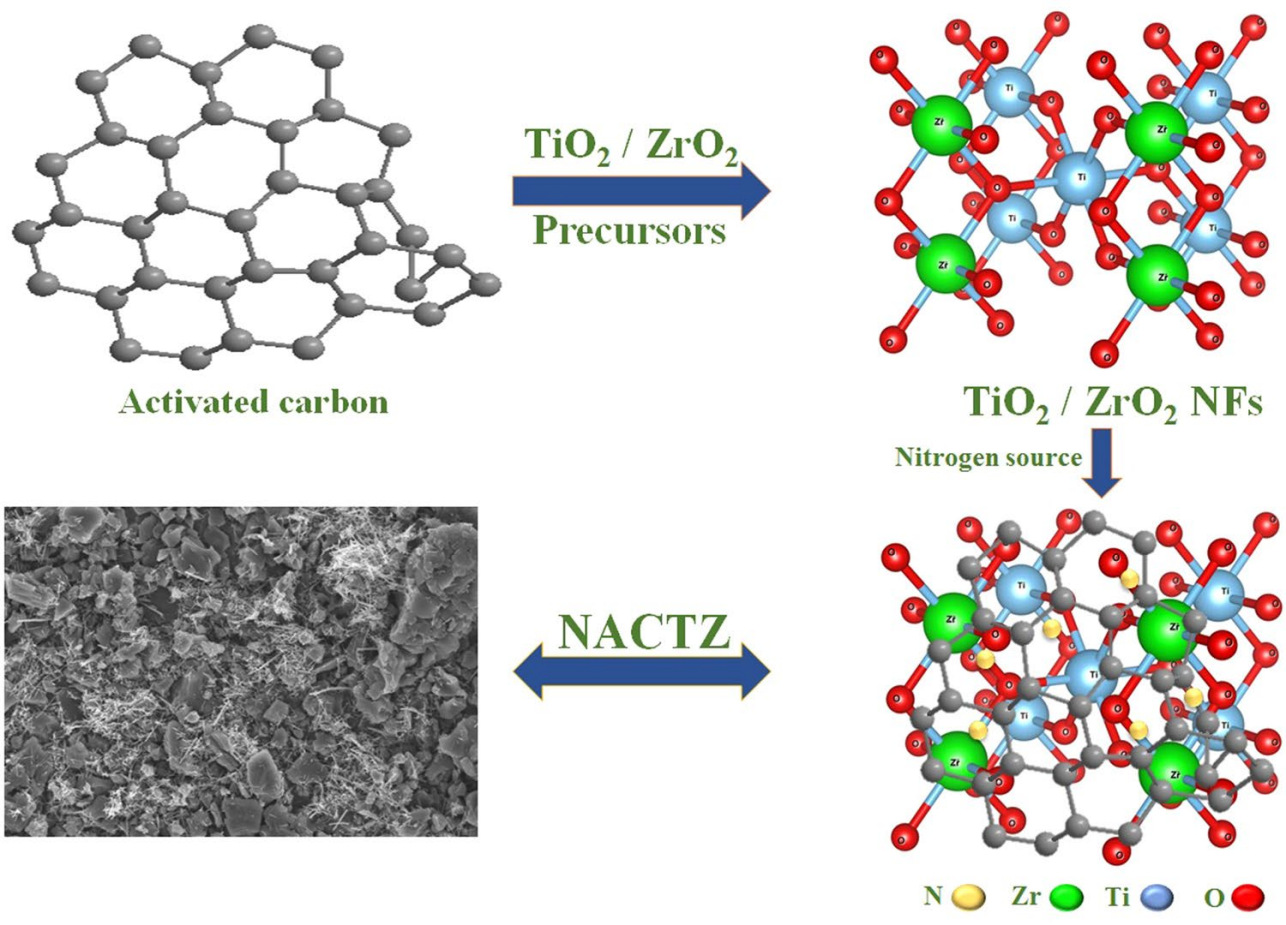
Schematic illustration of the synthesis of NACTZ nanocomposite.

In the present investigation, we demonstrate the following: (1) for the first time, nitrogen doped AC/TiO_2_ and ZrO_2_ nanofibers (NFs) are introduced as novel electrode materials for the CDI; (2) an effective strategy for fabricating the novel electrode is described, based on morphology and structure, as displayed in Fig. 1. The introduced composite has been prepared by using the electrospinning and hydrothermal processes. Electrospinning, as a fascinating technique to produce nanofibers with superb performance over the other nanostructures, was utilized for the fabrication of ZrO_2_ incorporated TiO_2_ NFs by calcination of electrospun mats composed of zirconium isopropoxyl, titanium isopropoxyl and polyvinyl acetic acid. Afterwards nitrogen and activated carbon were introduced to TiO_2_/ZrO_2_ by a hydrothermal treatment. The designed electrode configurations exhibit effective nitrogen doping and high electrochemical performance.

## Results and Discussion

### Phase morphology

**Figure 2:**
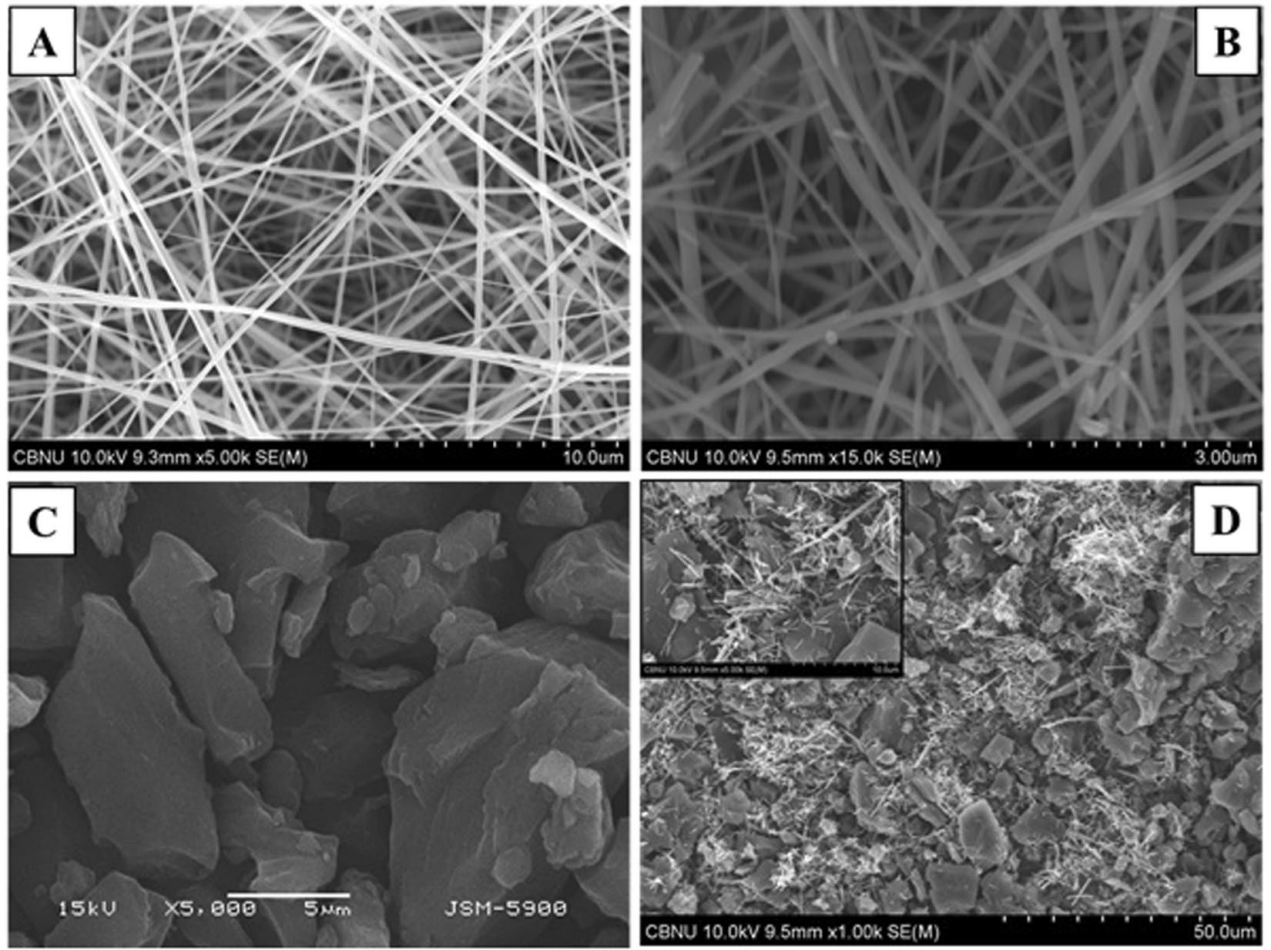
(**A** and **B**) FESEM image of the prepared fibers after electrospinning without calcination; and FE-SEM image of the obtained powder after calcination; (**C**) SEM image of pristine activated carbon, and (**D**) FESEM images of NACTZ (inset is high magnification).

Currently, it is widely known that the electrospinning process has many advantages over the other various nanofiber producing techniques, which are attributed to its ability for slurries spinning as well as its effectiveness and high yield^37^. Figure 2 shows the morphology of the synthesized TiO_2_/ZrO_2_ NFs and the morphology of the NACTZ composite which were investigated by FESEM imagery. Figure 2A shows the morphology of the electrospun mat contained entwined zirconium n-propoxyl and titanium iso-propoxyl fibers; the obtained nanofibers are smooth, continuous, and bead-free, and ranging from 100 to 600 nm in diameter. Figure 2B shows that after the hydrothermal process, the produced nanofibers were not harshly affected, which is attributed to the polycondensation tendency of the used metal alkoxides precursors^38^. However, a few broken nanofibers may be observed, but the axial ratio is still large, which reflects the preservation of the main advantage of the nanofibrous morphology. Figure 2C shows the SEM imagery of the pristine AC, which reveals a rock-like morphology and a heterogeneous and irregular surface. Figure 2D shows the low magnification FESEM imagery for NACTZ nanocomposite, while the inset image shows a high magnification. The rough surface of the synthesized NACTZ nanocomposite is evident, as are the TiO_2_/ZrO_2_ NFs that are highly dispersed on AC granules, and filling the cracks between AC particles without blocking its pores.

**Figure 3:**
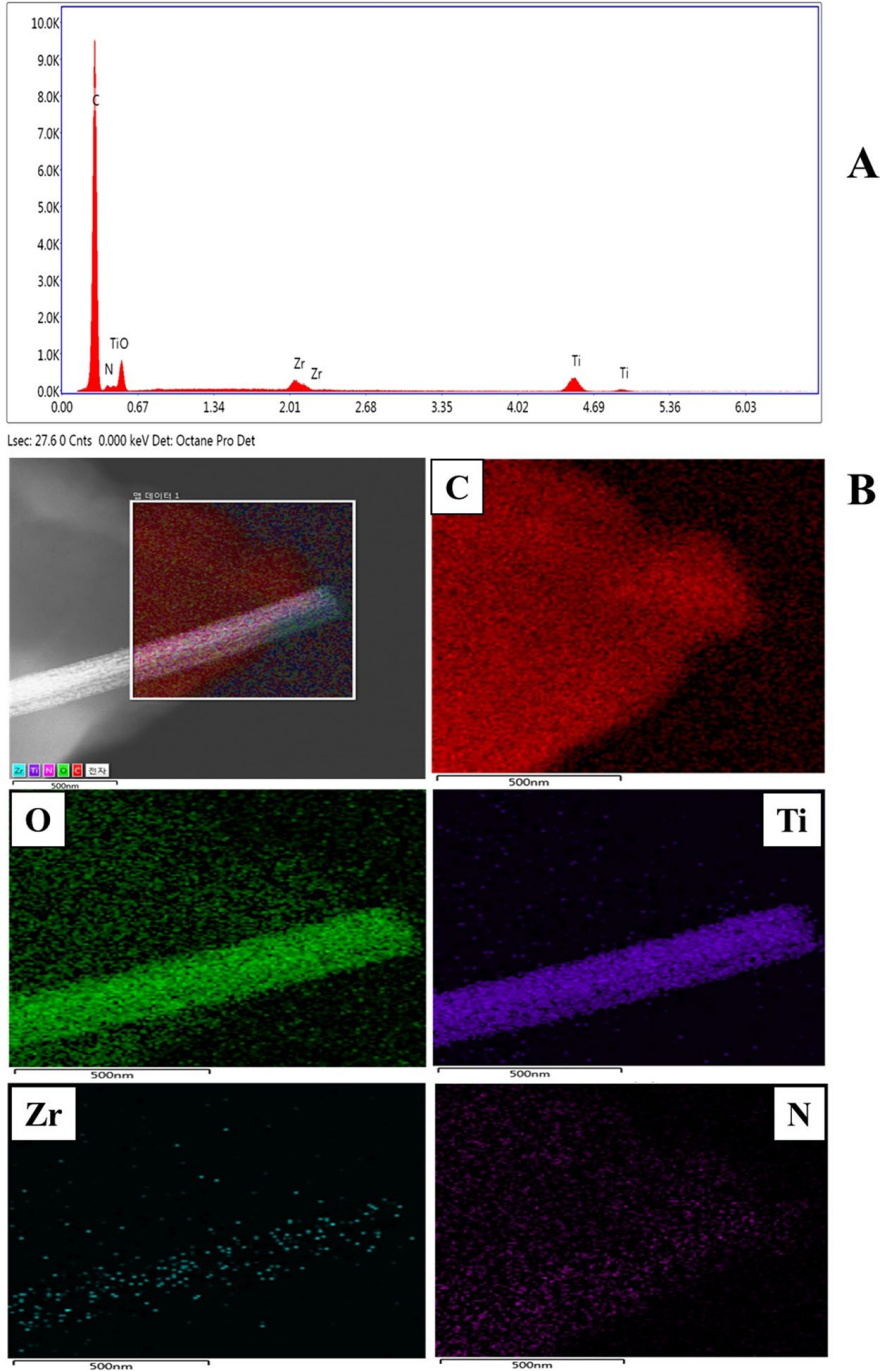
(**A**) FE SEM-EDX, and (**B**) TEM mapping of the NACTZ nanocomposite

To further understand the distribution of the nanofibers along with the activated carbon in the NACTZ nanocomposite, FE-SEM-EDX and TEM mapping analyses were carried out. Figure 3A shows the typical EDX spectrum of NACTZ. The analysis displayed the existence of carbon (C), oxygen (O), titanium (Ti), zirconium (Zr) and nitrogen (N) with the wt% concentrations 84.41, 8.02, 3.94, 0.5 and 3.12, respectively, no other peaks related to impurities in the spectrum can be observed, which strongly affirms that the fabricated composite is purely made of C, O, Ti, Zr and N. The TEM-mapping analyses Fig. 3(B) show that O, Ti, Zr and N are well dispersed on the introduced nanocomposite while carbon (C) is the main element, which also confirms that the nanofibers are embedded inside the AC.

**Figure 4:**
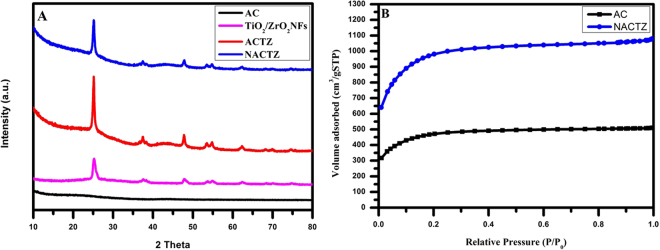
(**A**) XRD patterns for the synthesized electrode materials; pristine AC, TiO_2_/ZrO_2_ NFs, ACTZ and NACTZ, and (**B**) Nitrogen adsorption–desorption isotherms of the prepared materials.

XRD was carried out to investigate the crystal phase of the fabricated materials and further prove the successful preparation for NACTZ. Figure 4A shows the typical XRD spectrum of AC, TiO_2_/ZrO_2_ NFs, ACTZ and NACTZ. In the case of AC, two very weak and broad peaks near 26.3° and 42.2° can be observed, which are indexed to (0 0 2) and (1 0 0) for the planes of graphite, respectively [JCPDS card no. 41–1487]. For the TiO_2_/ZrO_2_ NFs, diffraction peaks are located at 25.35°, 36.88°, 37.78°, 38.5°, 48.07°, 53.92°, 55.11°, 62.07°, and 62.72°,which are attributed to the crystal planes (1 0 1), (1 0 3), (0 0 4), (1 1 2), (2 0 0), (1 0 5), (2 1 1), (2 1 3) and (2 0 4) for anatase TiO_2_ NFs, respectively [JCPDS card no. 00–004–0477]. No peaks for ZrO_2_ NFs can be observed because of its low doping percentage suggesting that ZrO_2_ NFs are homogeneously dissolved in the crystal structure of TiO_2_ NFs^39^. On the other hand, the ACTZ and NACTZ nanocomposite clearly reveals the same diffraction peaks of TiO_2_/ZrO_2_ NFs in addition to a broad peak between 20° and 25° which can be assigned to the (0 0 2) diffraction for the graphite crystallites, demonstrating that the TiO_2_/ZrO_2_ NFs incorporated successfully into AC. However, an observation displays that with nitrogen doping, the XRD peak intensities of anatase has become weaker as well as the width of the XRD diffraction peaks of anatase turns into slightly wider, demonstrating the reduce in the degree of crystallinity thus the formation of smaller TiO_2_ crystallites. Consequently, the fabricated NACTZ is compared with ACTZ based on the full width at half maximum (FWHM), d-spacing and crystal size according to the main peak at (1 0 1). The values of FWHM for NACTZ and ACTZ were calculated using the Scherrer equation (0.32 for NACTZ and 0.29 for ACTZ)^40^. Therefore, the crystal size was decreased from 27.84 nm to 25.05 nm due to nitrogen doping which may function as a growth inhibitor^41,42^.

The specific surface area of the fabricated materials were experimented by N_2_ adsorption desorption isotherms. Figure 4B displays the N_2_ sorption isotherms in addition to the pore size distribution plots of AC and NACTZ. Noticeably, it can be seen that AC displays typical type I isotherm and the hysteresis loop of NACTZ demonstrates an identical microporous structure for a carbon-based composite materials for CDI electrode. The NACTZ nanocomposite shows the highest specific surface area and the BET was estimated and found to be 3675.47 m^2^ g^−1^, whereas the specific surface area for the AC sample was estimated to be 1760 m^2^ g^−1^. It should be noted that the improvement in the specific surface areas of the NACTZ in comparison with the pristine AC could be attributed to the combination between the TiO_2_/ZrO_2_ NFs and nitrogen which can improve the porosity of NACTZ leading to increase the electrochemical characteristics and capacitive behavior as well.

**Figure 5:**
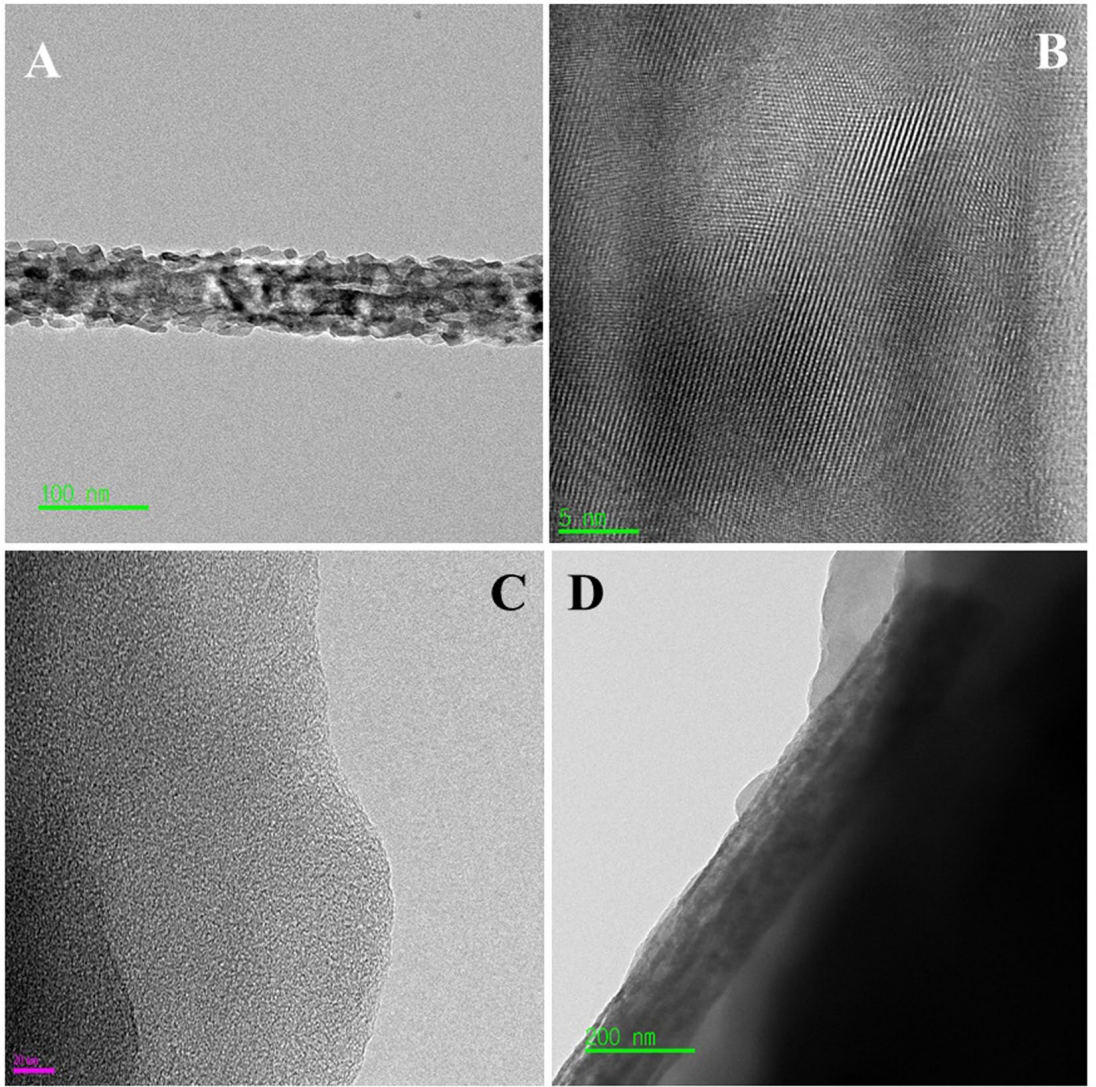
Normal TEM (**A**) and HR TEM image for the investigated fiber (**B**). Panels (**C**) and (**D**) display normal TEM images of the pristine activated carbon and NACTZ nanocomposite, respectively.

Figure 5 shows the morphological structure and crystallinity of TiO_2_/ZrO_2_ NFs and NACTZ which were investigated by TEM measurement. Figure 5A shows the normal TEM imagery for the TiO_2_/ZrO_2_ NFs; the diameter of the prepared fiber was calculated and the value is 72.2 nm. Additionally, Fig. 5B shows the HR-TEM imagery of the TiO_2_/ZrO_2_ NFs, which indicates the crystallinity of the sample through describing the atoms arrangement within the atomic plane. The lattice fringes of the investigated fiber possess an interplanar spacing of 0.361 nm which can be indexed to [1 0 1] plane of anatase TiO_2_ (the main content of the prepared fiber), which is complemented with XRD analysis. Figure 5C depicts the TEM imagery of the pristine AC. It can be seen that the surface of AC is rough, thin and shows a flat micro-plates. The TEM imagery for the NACTZ nanocomposite (Fig. 5D) reveals that the TiO_2_/ZrO_2_ NFs are superficially attached to the activated carbon.

**Figure 6:**
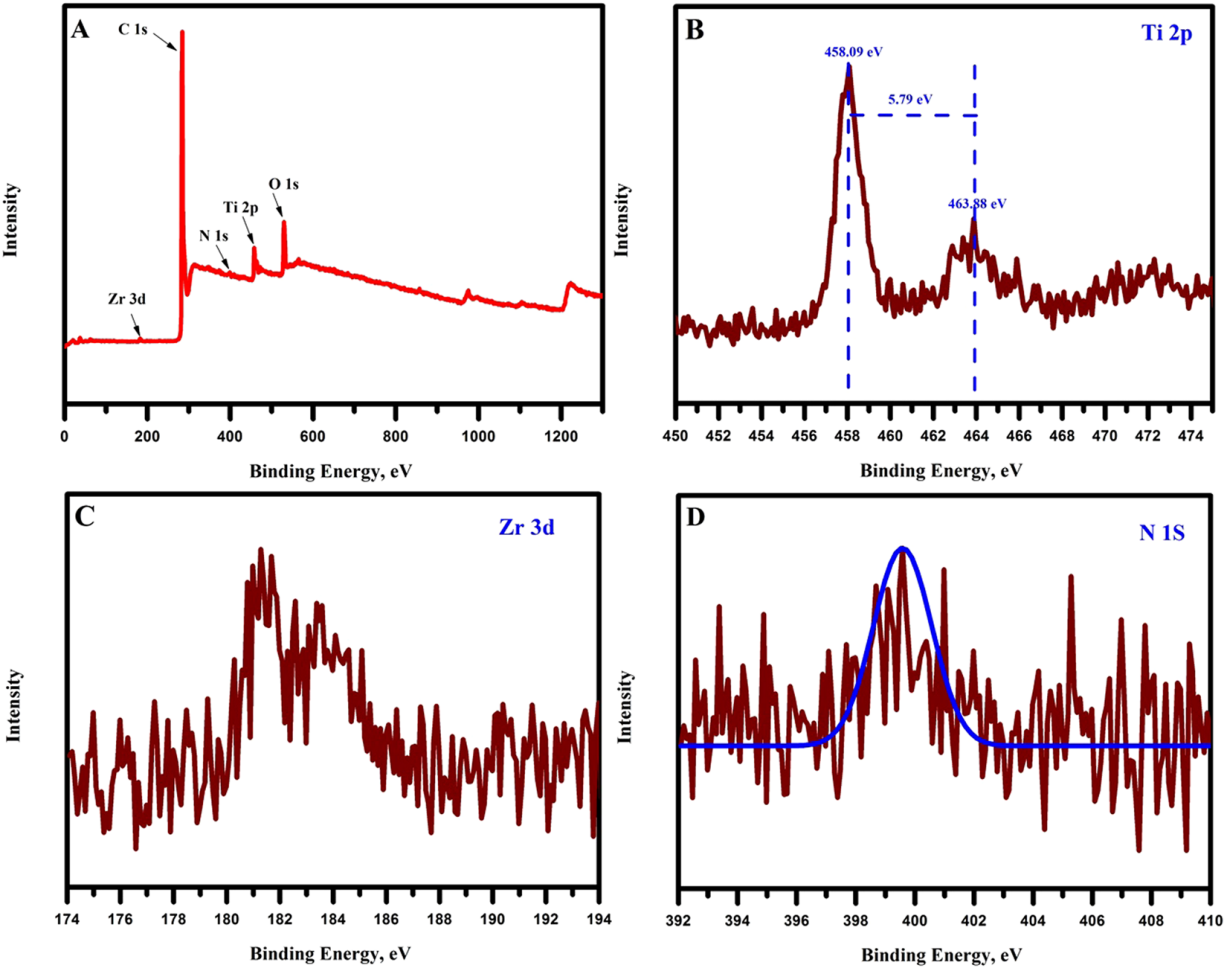
(**A**) XPS spectra survey of as-synthesized NACTZ nanocomposite (**B**) Ti 2p spectra (**C**) Zr 3d spectra (**D**) N 1 s spectra.

The chemical composition of the as-prepared NACTZ nanocomposite was investigated by X-ray photoelectron spectroscopy (XPS), which is considered to be a powerful tool that can be carried out to monitor the changes that occurred in the nanocomposite structure. Figure 6A depicts the XPS survey scan of the NACTZ nanocomposite. The XPS scan of the NACTZ revealed the presence of C, O, Ti, Zr and N which highly support and complement the TEM elemental mapping results. The Ti 2p spectra (Fig. 6B) show a pair of symmetrical peaks that appear at 458.09 and 463.88 eV, which are assigned to Ti 2P_3/2_ and Ti 2P_1/2_, respectively. Furthermore, the spin-orbital splitting between Ti 2P_1/2_ and Ti 2P_3/2_ of 5.79 eV reflects that the obtained Ti (IV) is a normal state in the synthesized TiO_2_/ZrO_2_ NFs^43^. From Fig. 6C, the XPS of Zr 3d_5/2_ electrons depicts the presence of peak at 181.46 eV which is consistent with the value reported for Zr (+IV) ions in the Zr-doped TiO_2_^44^. Additionally, the survey scan of NACTZ nanocomposite indicates the existence of N 1 s peak. The N 1 s spectrum (Fig. 6D) of the NACTZ was fitted into one peak at binding energy of 399.6 eV, which is assigned to the pyridinic N (N-6)^45^. This offers a solid proof that the NACTZ nanocomposite was successfully doped by nitrogen atoms. It is worth noting that the existence of pyridinic N (N-6) has advantages for CDI, such as increasing the pseudocapacitance and the wettability as well as improving the ion diffusion and transportation^46^.

**Figure 7:**
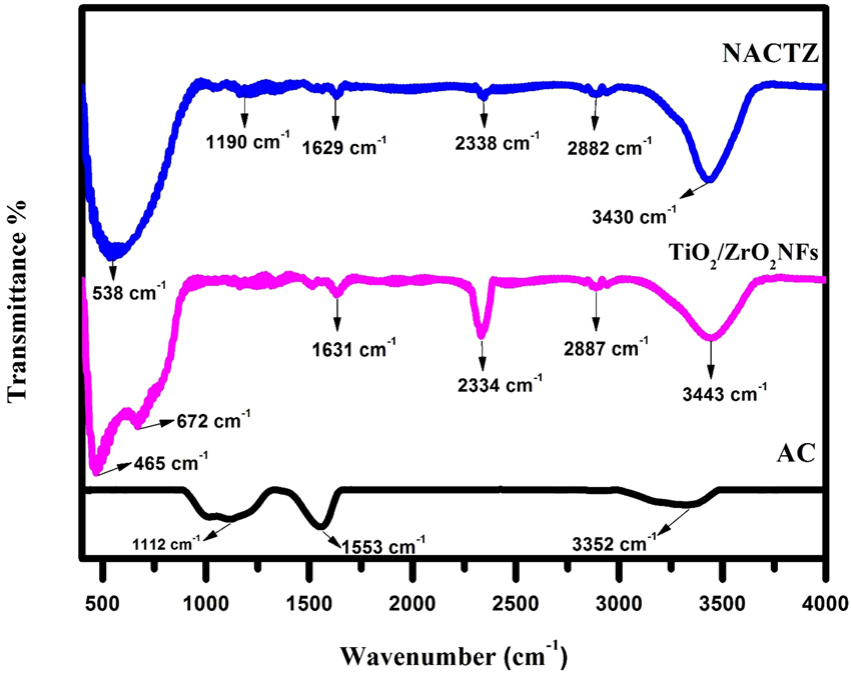
FTIR spectra of pristine AC, TiO_2_/ZrO_2_ NFs and NACTZ.

Figure 7 shows the FTIR spectrum, which was carried out to investigate the structural features of the AC, TiO_2_/ZrO_2_ NFs, and the significant changes that were observed in NACTZ after their interaction. The FTIR spectra of the AC show peaks at 1129 cm^−1^ (C‒O stretching vibration)^47^, 1560 cm^−1^ (C = C bonding in aromatic carbons)^48^ and 3324 cm^−1^ (OH group)^49^. On the other side, the vibration band at 465–672 cm^−1^ in the TiO_2_/ZrO_2_ NFs was attributed to a Ti–O vibration and the bands at 3443, 2887, 2334 and 1631 cm^−1^ were assigned to O–H bending of the absorbed water molecules, C–H stretch, O = C = O and C = C (skeletal vibration of unoxidized graphitic domains), respectively^50,51^. In the case of the FTIR spectra of the NACTZ, the existence of all the peaks corresponding to TiO_2_/ZrO_2_ NFs can be seen, but with small shifting of peak positions that occurred, which is attributed to the interaction between TiO_2_/ZrO_2_ NFs and activated carbon. NACTZ has a characteristic band at about 538 cm^−1^ that can be assigned to Ti–O vibration and the characteristic peaks around 2882 cm^−1^ and 2338 cm^−1^ correspond to the C–H stretch and O = C = O. Meanwhile, the broad band at 3430 cm^−1^ was related to either ‒OH and/or ‒NH moieties that were detected as well as the band at 1190 cm^−1^ corresponding to the C–N stretching, suggesting that nitrogen atoms are incorporated into activated carbon and TiO_2_/ZrO_2_ NFs^52,53^.

### Surface wettability test

**Figure 8:**
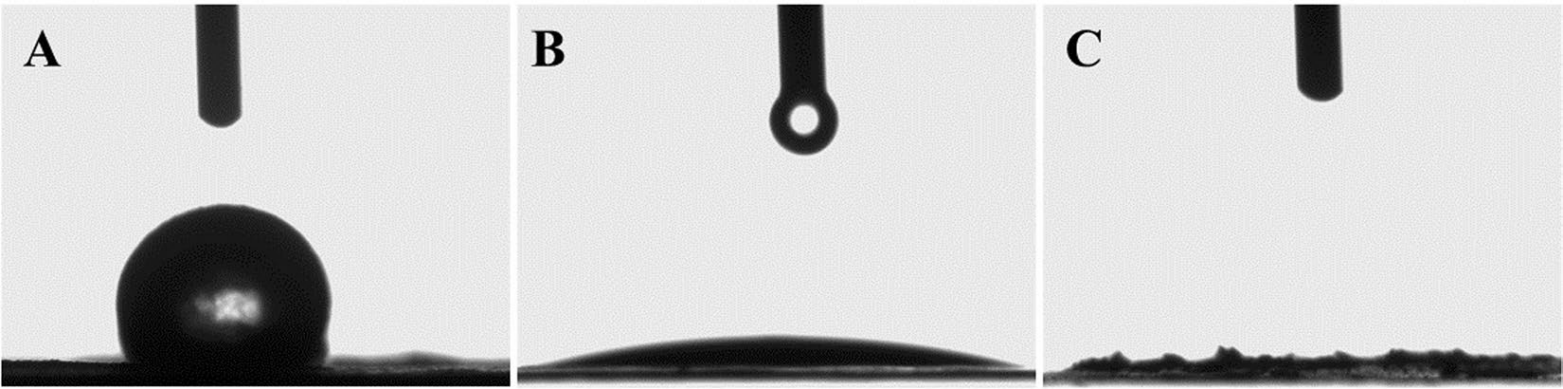
Optical micrographs of the water contact angles on the surface of the fabricated electrode (**A**) AC, (**B**) ACTZ and NACTZ (**C**).

It is well known that the surface wettability is a key factor for increasing the electrode adsorption capacity. Whereas, the main dilemma for all carbon electrodes, including activated carbon, is their low surface wettability based on their hydrophobicity. Herein, the activated carbon was by doping with TiO_2_/ZrO_2_ NFs and nitrogen to enhance the hydrophilicity of the AC electrode as well as the wettability. The hydrophilic characteristics of the doped and un-doped electrodes, which were provided by measurement of the static water contact angles at the synthesized electrode surface as shown in Fig. 8. Doping the electrode significantly enhanced the surface wettability of AC, the corresponding contact angle of the pristine AC was about 148° (Fig. 8A). In contrast, Fig. 8B shows that the AC electrode doped by TiO_2_/ZrO_2_ NFs revealed highly hydrophilic performance with 16.9° water contact angle. Consequently, after doping the AC by nitrogen and TiO_2_/ZrO_2_ NFs, the electrode demonstrates extremely higher hydrophilic property than AC and ACTZ electrodes, the entire contact angle of NACTZ electrode reaching to 3° (Fig. 8C).

### Electrochemical behavior

**Figure 9:**
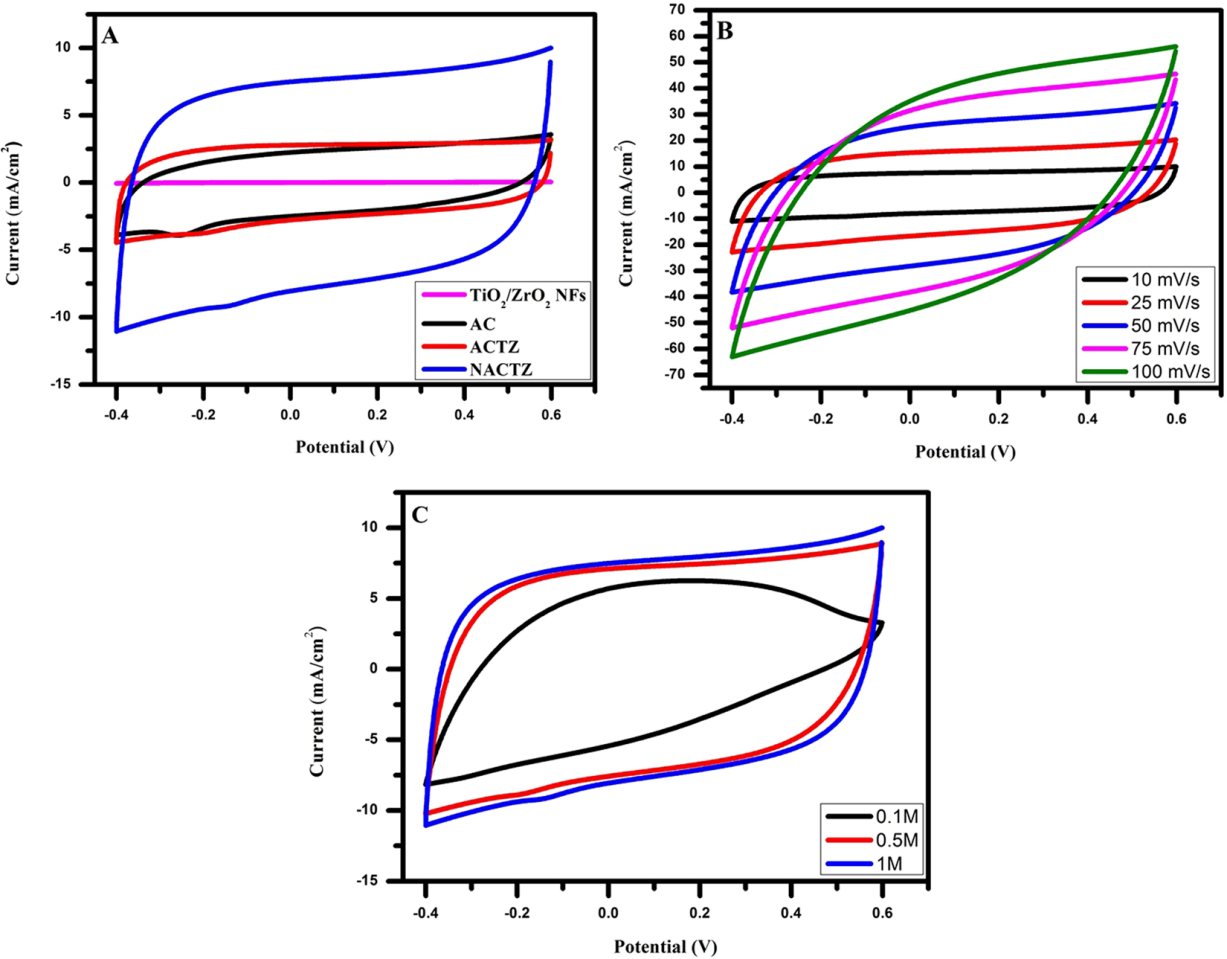
(**A**) Cyclic voltamogramms (CV) for the fabricated electrodes at a scan rate of 10 mVs^−1^ in a 1 M NaCl aqueous solution; (**B**) for the NACTZ electrode at various scan rates in a 1 M NaCl aqueous solution; and (**C**) comparative CV curves of NACTZ electrode at various NaCl concentrations at a scan rate of 10 mV s^−1^.

The electrochemical measurements for the fabricated electrodes were carried out in a NaCl aqueous solution to investigate the influence of nitrogen and TiO_2_/ZrO_2_ NFs on AC. Figure 9A reveals the cyclic voltammetry (CV) curves of the TiO_2_/ZrO_2_ NFs, AC, ACTZ and NACTZ electrodes in 1 M NaCl at 10 mV s^−1^. It can be observed that the all CV curves almost exhibits a quasi-rectangular shape, besides the absence of faradic peaks in the working potential of −0.4 to 0.6 V, suggesting that the capacitive behavior be attributed to the electric double-layer formation because of the coulombic interactions instead of redox reactions^54^. Moreover, the curves reveal a good symmetry, indicating a quite reversible capacitive performance^55^. Obviously, the NACTZ electrode demonstrates greater improvement in electrochemical performance compared to TiO_2_/ZrO_2_ NFs, AC and ACTZ electrodes which is attributed to the co-doping of nitrogen and TiO_2_/ZrO_2_ NFs which improved the hydrophilicity and the surface wettability of NACTZ as well as the porous nature resulting in the enhancement of the speed up of the ion transfer into the electrolyte to achieve the electric double-layer property^37^. The CV humps of the NACTZ electrode at different sweep rates are shown in Fig. 9(B). It can be seen that when the scan rate increases for NACTZ electrode the CV curves converted gradually from rectangular shape to leaf-like shape according to the inherent resistivity resulting from the salt solution, additionally it cannot be seen a redox peaks suggesting a significant capacitive behavior of NACTZ as CDI electrode material. Figure 9C displays the CV curves for NACTZ at NaCl in different concentrations. Clearly, the specific capacitances increased when the concentration of NaCl increased reflecting excellent electrosorption capacity in addition to hard saturation.

**Figure 10:**
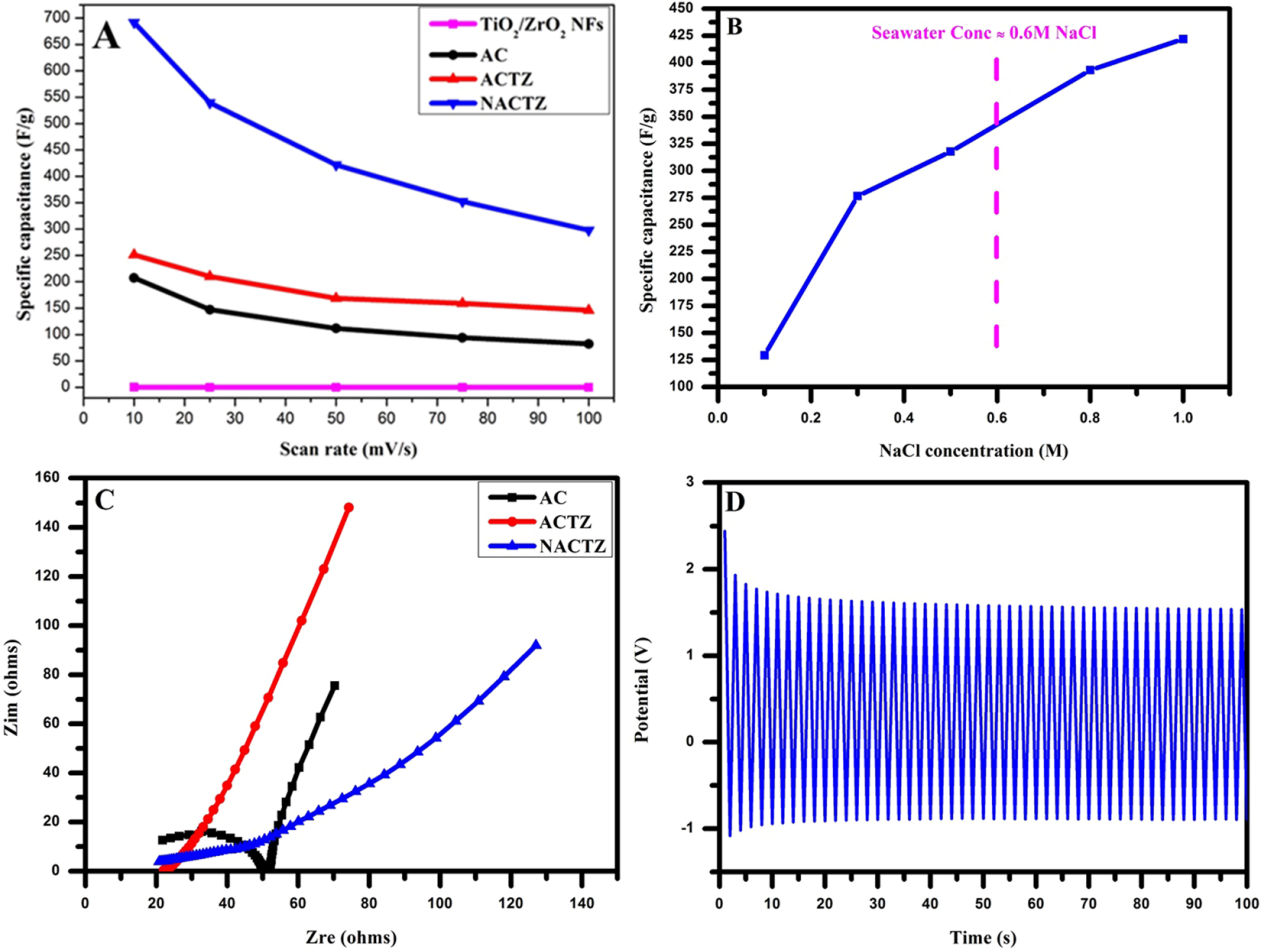
(**A**) Specific capacitance for the obtained electrode materials, (**B**) influence of NaCl concentration on the NACTZ performance, (**C**) Nyquist plot for the proposed electrode materials and (**D**) the continuous GC profile of the NACTZ electrode at a constant current load of 0.1 A g ^−1^.

Figure 10A, compares the specific capacitance of TiO_2_/ZrO_2_ NFs, AC, ACTZ and NACTZ electrodes which is calculated according to Eq. (1) at various scan rates. It is important to note that the area under the CV curve of NACTZ electrode is much larger in comparison with those of TiO_2_/ZrO_2_ NFs, AC and ACTZ, suggesting the higher specific capacitance of the NACTZ electrode. It can be seen the obvious decay in specific capacitance at high scan rates, and vice versa the increased of specific capacitance at low scan rates. This finding occurs, because at low scan rates, the ions obtain adequate time to transport into the NACTZ composite, resulting in the extreme ions migration reaction becoming possible, thus leading to distinct capacitive behavior at low scan rates, compared to the high scan rates. Apparently, the NACTZ electrode permanently exhibits the highest value at any selected sweep rate compared with the other electrodes. Therefore, NACTZ could be fabricated and used as a conductive electrode for the capacitive deionization process. The specific capacitance at 10 mV s^−1^ of the NACTZ electrode is 691.78 F g^−1^, which is much higher than those of ACTZ (251.32 F g^−1^), AC (207.46 F g^−1^) and TiO_2_/ZrO_2_ NFs (0.4 F g^−1^). Comprehensively, the enhancements in specific capacitances are essentially according to the co-doping of nitrogen and TiO_2_/ZrO_2_ NFs bringing: (i) increased charge transfer; (ii) improved specific surface area thus the pore volume; and (iii) enhance the hydrophilicity and electronic conductivity^56^. As displayed in Fig. 10B the greatly interest finding achieved in this study; that the NACTZ electrode could be successfully used for water desalination because the salt removal exceeds the threshold of seawater concentration.

EIS measurements were employed to investigate the electrical conductivity of the fabricated electrodes. Figure 10C introduces the EIS of AC, ACTZ and NACTZ electrodes in 1 M NaCl solution. The Nyquist profiles exhibiting that all synthesized electrode materials possess the same shapes, involving a quasi-semicircle in the high frequency region as well as a nearly vertical line in the low frequency region. The intersection at the real axis (Z′) indexed to the equivalent series resistance (ESR), whereas the width of the semicircle refers to the charge transfer resistance (Rct) and double-layer capacitance (Cdl)^57^. It can be seen, according to the diameter of the semicircle that the Rct of NACTZ is much lower than those of AC and ACTZ, reflecting the enhancement in the charge transfer ability of NACTZ. This also matches the CV results and the specific capacitance value of NACTZ being the highest. Moreover, it can be claimed that the capacitive behavior of carbon-based electrodes could be improved by nitrogen doping.

The cycle characteristic of the NACTZ nanocomposite was also tested by utilizing the continuous GC measurement in 1 M NaCl aqueous solution through a 0.1 A g^−1^ current density. It can be seen in Fig. 10D, that the profile exhibits a triangular shape with linear lines, suggesting that the capacitive behavior occurs according to the formation of electrical double-layer rather than Faradaic reaction^58^. Additionally, no observed charge/discharge decay over 50 cycles, reflecting a high reversibility and cyclability for the NACTZ electrode. These important feature point to a long service life for the NACTZ electrode in CDI process.

### CDI behavior

**Figure 11:**
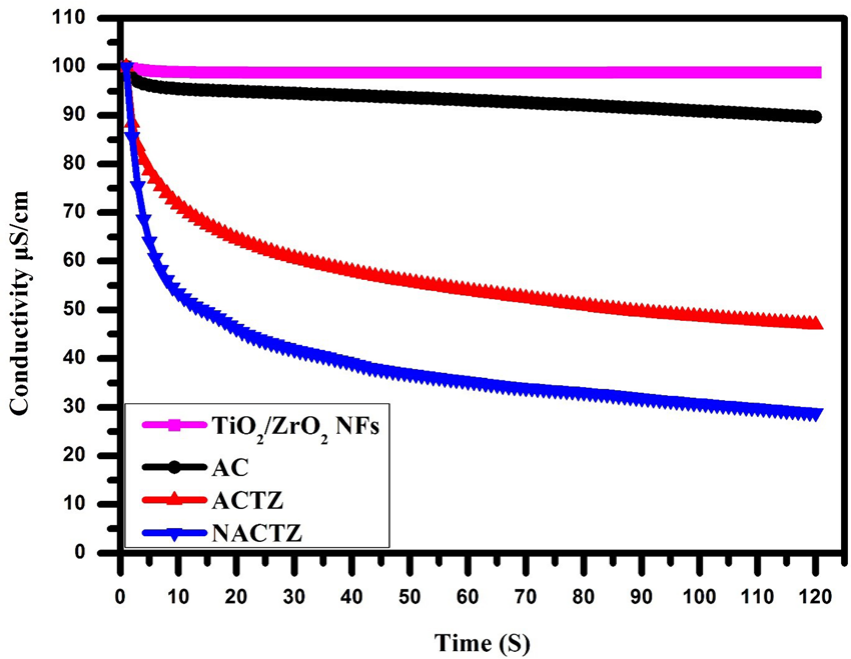
CDI performance for the fabricated electrodes in NaCl solution.

The deionization efficiency of the synthesized electrodes was plotted in Fig. 11, in NaCl aqueous solution having an initial concentration of ~100 μS cm^–1^ through a constant operational voltage of 1.2 V. It can be clearly seen that once the electric field is applied the solution conductivity at the early stage starts to quickly decrease, implying the fast transportation of the salt ions. Afterwards, the change in solution conductivity persistently attenuates until reaching adsorption equilibrium, because of the electrodes becoming saturated as well as the electrostatic repulsion occurring between the electrode and electrolyte ions. Clearly, the NACTZ nanocomposite electrode demonstrates the largest adsorption capacity in comparison to ACTZ, AC and TiO_2_/ZrO_2_ NFs. According to Eq. (2), the salt removal efficiencies (η) of AC, ACTZ, and NACTZ electrodes are 25.44, 53.08 and 71.19%, respectively. As expected, NACTZ exhibits the best salt removal efficiency. It should be noted that when the saturation time increased the adsorption of ions increased on the surface of the electrode. Hence, according to Eq. (3) the electrosorptive capacity of the NACTZ is estimated to be 3.98 mg/g which shows a remarkable improvement in comparison to ACTZ (2.96 mg/g), the pristine AC (1.4 mg/g) and TiO_2_/ZrO_2_ NFs (0.067 mg/g). Thus, the desalination performance could be ordered as: NACTZ > ACTZ > AC > TiO_2_/ZrO_2_ NFs. The NACTZ nanocomposite exhibiting the highest desalination performance can be attributed to: (1) the nitrogen doping enhancibg the specific surface area and that can influence the adsorption of ions by providing more sites; (2) highly hydrophilic performance; (3) reduced the expulsion of co-ion; (4) and its concentration polarization is low.

**Figure 12:**
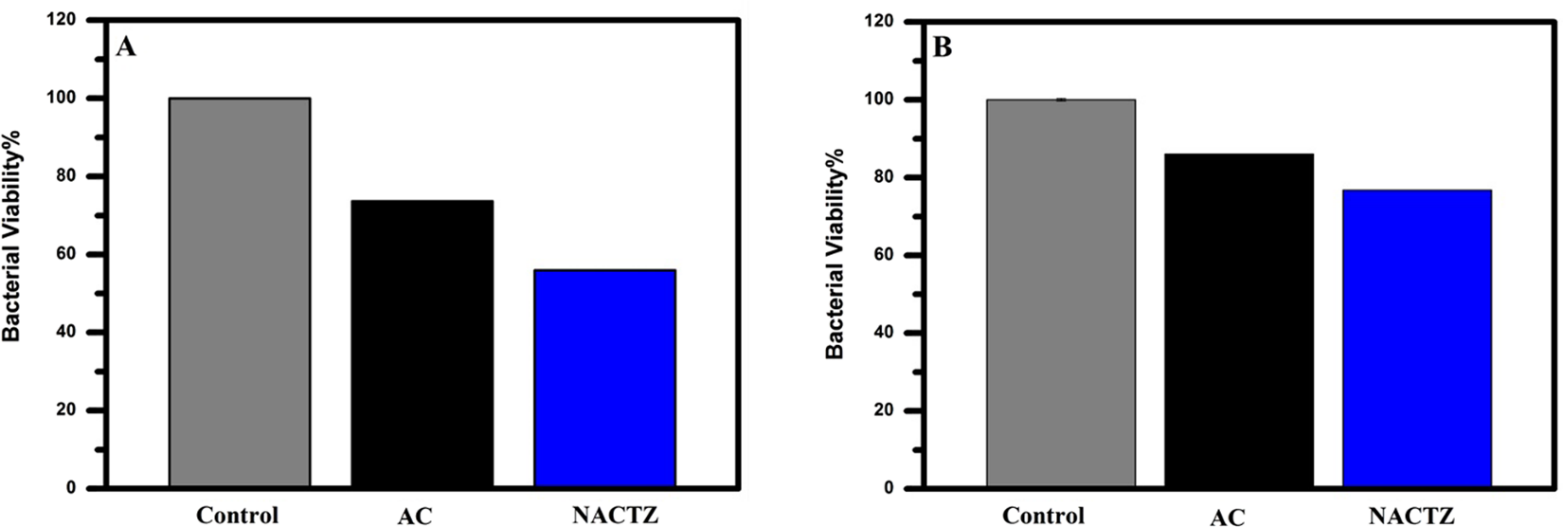
Viability of *E. coli* (**A**) and S. aureus (**B**) after 24 h treated on different samples.Table 1: The specific capacitance and electrosorption capacity for different carbon-based electrodes.

Our strategy through this work was not only to obtain the requirements needed for CDI electrode materials and enhanced the desalination performance but also demonstrates a high competence for effective disinfection via fabricating the CDI electrode with materials having high antimicrobial activity. In order to evaluate the antibacterial performance of the pristine AC and NACTZ against E. coli and S. aureus, spectrophotometric analysis was recorded. As displayed in Fig. 12A and B, shows that the pristine AC, does not demonstrate any considerable efficiency in its antibacterial activity towards Gram-positive or further Gram-negative bacteria. On the other side, in comparison with AC, the NACTZ nanocomposite exhibits a robust antibacterial performance and high killing efficiency towards E. coli and S. aureus in spite of the high injection of bacteria concentration being 10^7^ CFU mL^−1^. As postulated, NACTZ electrode exhibits the highest antimicrobial performance, which could be assigned to the TiO_2_ and ZrO_2_-doping which both possess a robust antibacterial activity towards a broad spectrum of bacteria^38,59^. TiO_2_ and ZrO_2_ have the ability for killing Gram-negative and Gram-positive bacteria, despite the lowest sensitivity of Gram-positive bacteria according to their capability for the formation of spores^60^. Moreover, the antibacterial activity for TiO_2_ is widely known, which is attributed to its ability to produce the reactive oxygen species (ROS), specifically the formation of hydroxyl free radicals as well as peroxide within UV-A irradiation^61^. Additionally, it is recently reported that nano-sized TiO_2_ has the ability for killing several viruses such as hepatitis B virus^62^, MS2 bacteriophage^63^ and Herpes simplex virus^64^. Therefore, it can be claimed that the NACTZ nanocomposite is a promising candidate as disinfectant for water.

In summary, Table 1 lists the reported data on the specific capacitance and electrosorption capacity for different carbon-based electrodes, to further prove that NACTZ is beneficial to improving the CDI performance.

**Table 1:** The specific capacitance and electrosorption capacity for different carbon-based electrodes.

Electrode material	Specific capacitance (Fg^−1^)/scan rate (mV s^−1^)	Applied voltage (V)	Initial concentration (mg L^−1^)	Electrosorption capacity (mg g^−1^)	Ref.
MC	251/1	1.2	25	0.68	^68^
CNTs-MC	132.6/10	1.2	40	0.69	^69^
RG-CNTs	175/1	2	~27	1.41	^70^
AC	169/1	1.2	~25	0.25	^68^
RG-AC	181/1	1.2	~500	2.94	^71^
AC-MnO_2_	77.2/10	1.2	~25	0.99	^72^
AC/TiO_2_ NPs	**—**	1.2	500	2.7	^73^
AC	207.4/10	1.2	~50	1.4	This work
ACTZ	251.3/10	1.2	~50	2.96	This work
NACTZ	691.7/10	1.2	~50	3.98	This work

## Conclusions

NACTZ is rationally designed and obtained by two steps: an electrospinning technique followed by a hydrothermal treatment and then utilized as CDI electrode material for the first time. TiO_2_/ZrO_2_ NFs incorporation and nitrogen doping strongly improved the performance of the pristine activated carbon in the CDI process. Impressively, with respect to the most important parameters that could achieve high desalination behavior, wettability, specific capacitance and electrosorption capacity, the NACTZ hybrid networks demonstrates high hydrophilicity with lower contact angle of 3°, large specific capacitance of 691.78 F g^−1^, and increased electrosorpotive capacity of 3.98 mg g^−1^. Additionally, the NACTZ nanocomposite demonstrates high disinfection capacity against Gram-positive and Gram-negative bacteria. It is postulated that the introduction of TiO_2_/ZrO_2_ NFs and N co-doped AC will not only open new avenue for promising way to create a novel high performance CDI electrode materials, but may also open opportunities to be applied for energy and electrochemistry application.

## Experimental

### Materials

Titanium (IV) isopropoxide (>97%, Sigma-Aldrich), N,N-dimethylformamide (DMF, 99.5%, Sigma-Aldrich), zirconium(IV) isopropoxide (Zr(Iso)) solution 70 wt% in 1-propanol Zr(OCH_2_CH_2_CH_3_)_4_(Sigma-Aldrich), poly(vinyl acetate) (PVAc, M.wt = 500,000 g/mol), and glacial acetic acid (Sigma-Aldrich), Activated carbon powder (CEP-21K, PCT Co., Korea, BET surface area = 2110 m^2^ g^−1^) and urea (Alfa Aesar) were obtained.

### Synthesis of Zr-doped TiO_2_ nanofibers (TiO_2_/ZrO_2_ NFs)

A sol–gel composed of Ti(Iso), Zr(Iso) and PVAc was prepared as follows: (titanium (Iso); 3.6 g and Zirconium (Iso); 0.28 g) was added to PVAc (14 wt.%, in DMF) solution, and then a few drops of CH_3_COOH acid were added until the solution became clear and get see-through. The solution was continuous mixed under stirred conditions for two hours. The achieved sol–gel was supplied to the electrospinning process at a high voltage of 18 kV using DC power supply (CPS-60 K02V1, Chung EMT Co., South Korea) at room temperature with 60% relative humidity. The distance was kept constant at 15 cm between tip and rotating cylinder. The ready electrospun nanofibers were collected then vacuously dried under vacuum for one day at 60 °C. After that, the nanofibers were calcined in air atmosphere at 600 °C for 1 h at the rate of 5 °C/min.

### Fabrication of nitrogen-doped activated carbon incorporated TiO_2_/ZrO_2_ NFs (NACTZ)

The prepared TiO_2_/ZrO_2_ NFs (0.06 g) were stirred and sonicated for 1 h in 100 ml de-ionized water under magnetic stirring, then a mix of 0.5 g amount of activated carbon and 0.5 g amount of urea was added to the solution, which has kept sonicated for 30 min. Later, the dispersed solution was subjected to the hydrothermal process in a Teflon-lined autoclave at 120 °C for 24 h. Finally, the solution was filtered and washed five times with distilled water and dried at 70 °C under vacuum oven. Activated carbon incorporated TiO_2_/ZrO_2_ NFs (ACTZ) was fabricated by the same strategy in urea-free solution.

### Characterization

The crystallinity was studied by X-ray diffractometer (XRD, Rigaku, Japan) with Cu Kα (λ = 1.54056 Å) radiation over Bragg angle 2θ ranging from 10° to100°. X-ray Photoelectron Spectroscopy analysis (XPS) (AXIS-NOVA, Kratos analytical Ltd., UK) was conducted to investigate the elemental and chemical compositions of the prepared material. The surface morphology of the samples was investigated via field emission transmission electron microscopy (FESEM) (FE-SEM; JEOL JEM-2200 FS, Japan). The particle sizes and shapes were examined through high resolution transmission electron microscopy (HR-TEM), scanning TEM (STEM) with elemental mapping and energy dispersive X-ray analysis (JEM-2200 FS, JEOL Ltd., Japan). The specific surface areas of the introduced materials, were estimated by the Brunauer–Emmett–Teller (BET) method of nitrogen sorption at 77 K by utilizing a Micromeritics Tristar 3000 analyzer. Fourier transform infrared (FT-IR) spectra of the samples were investigated with Spectrum 100 FT-IR, PerkinElmer, USA. Cyclic voltammetry (CV) experiments were tested by utilizing a VersaStat4 potentiostat instrument, as reported previously in our work^32^. The specific capacity can be calculated by integrating the full CV cycle to determine the average value according to the following relationship^65^:

1 $${\rm{Cs}}=\frac{\int i\,{dV}}{2{v}{\rm{\Delta }}{Vm}}$$

where, Cs is the specific capacitance (Fg^−1^), i is the response current (A), V is the potential (V), υ is the potential scan rate (Vs^−1^), and m is the mass of the electro-active materials in the electrode (g).

### Electrosorptive capacity measurement

The CDI electrodes were prepared as follows: the fabricated active materials and polytetrafluoroethylene (PTFE) binder were mixed in a weight ratio of 80:20 (active materials: PTFE) to investigate the electrosorptive capacity for the CDI system via the introduced carbon material. Each working electrode was then drop-casted by the mixed slurry on the carbon electrode, then subjected to a vacuum oven at 100 °C for 12 hours. The CDI electrode was obtained in a geometric surface of 40 mg/cm^2^. The salt removal efficiency (η) and electrosorptive capacity (Sc) of the electrode can be calculated according to the following equations^66^:

2 $${\rm{\eta }}=(\frac{Co-C}{Co})\times 100 \% $$

3 $${\rm{Sc}}=(Co-C)\,V/m$$

where, *Co* and *C* (mg/L) are the initial and final concentrations, respectively, *V* (L) is the total volume of the NaCl aqueous solutions, and *m* (g) represents the mass of the active components in the working electrodes.

### Antibacterial activity measurement

The antibacterial performance of N-AC/TZ nanocomposite was quantitatively evaluated on both Gram-positive and Gram-negative bacteria. The bacteria utilized in this work were Escherichia coli (ATCC 52922) and Staphylococcus aureus (ATCC 29231) as model organisms. The antibacterial properties of fabricated materials against each strain were determined by spectrophotometric method as described in ref.^67^.
